# Public Opinion and Sentiment Before and at the Beginning of COVID-19 Vaccinations in Japan: Twitter Analysis

**DOI:** 10.2196/32335

**Published:** 2022-05-09

**Authors:** Qian Niu, Junyu Liu, Masaya Kato, Yuki Shinohara, Natsuki Matsumura, Tomoki Aoyama, Momoko Nagai-Tanima

**Affiliations:** 1 Department of Human Health Sciences Graduate School of Medicine Kyoto University Kyoto Japan; 2 Department of Intelligence Science and Technology Graduate School of Informatics Kyoto University Kyoto Japan

**Keywords:** COVID-19, Japan, vaccine, Twitter, sentiment, latent dirichlet allocation, natural language processing

## Abstract

**Background:**

COVID-19 vaccines are considered one of the most effective ways for containing the COVID-19 pandemic, but Japan lagged behind other countries in vaccination in the early stages. A deeper understanding of the slow progress of vaccination in Japan can be instructive for COVID-19 booster vaccination and vaccinations during future pandemics.

**Objective:**

This retrospective study aims to analyze the slow progress of early-stage vaccination in Japan by exploring opinions and sentiment toward the COVID-19 vaccine in Japanese tweets before and at the beginning of vaccination.

**Methods:**

We collected 144,101 Japanese tweets containing COVID-19 vaccine-related keywords between August 1, 2020, and June 30, 2021. We visualized the trend of the tweets and sentiments and identified the critical events that may have triggered the surges. Correlations between sentiments and the daily infection, death, and vaccination cases were calculated. The latent dirichlet allocation model was applied to identify topics of negative tweets from the beginning of vaccination. We also conducted an analysis of vaccine brands (Pfizer, Moderna, AstraZeneca) approved in Japan.

**Results:**

The daily number of tweets continued with accelerating growth after the start of large-scale vaccinations in Japan. The sentiments of around 85% of the tweets were neutral, and negative sentiment overwhelmed the positive sentiment in the other tweets. We identified 6 public-concerned topics related to the negative sentiment at the beginning of the vaccination process. Among the vaccines from the 3 manufacturers, the attitude toward Moderna was the most positive, and the attitude toward AstraZeneca was the most negative.

**Conclusions:**

Negative sentiment toward vaccines dominated positive sentiment in Japan, and the concerns about side effects might have outweighed fears of infection at the beginning of the vaccination process. Topic modeling on negative tweets indicated that the government and policy makers should take prompt actions in building a safe and convenient vaccine reservation and rollout system, which requires both flexibility of the medical care system and the acceleration of digitalization in Japan. The public showed different attitudes toward vaccine brands. Policy makers should provide more evidence about the effectiveness and safety of vaccines and rebut fake news to build vaccine confidence.

## Introduction

A novel coronavirus causing COVID-19 was first identified in December 2019 [1]. As of February 24, 2022, the cumulative confirmed cases and deaths were approximately 426 million and 5.9 million, respectively, globally [2], of which 4,607,029 confirmed cases and 22,272 death cases were reported in Japan [3]. As of February 24, 2022, Japan had suffered 5 waves of infection and, as of this writing, was going through the sixth wave. During the fifth wave, the Tokyo Olympic Games were held, and infection cases had surged to 1,556,998 when the Games finished. The sixth wave began after the first local case of the Omicron variant and soon broke the records of cases and deaths in the fifth wave. The pandemic has had a significant adverse effect on individuals, governments, and the economy in Japan [4].

To end the worldwide pandemic, several COVID-19 vaccines were developed, and large-scale vaccination was called for to protect people across the world [5-7]. Japan’s vaccination campaign was slower in the early stages than in other developed countries. Japan experienced significant delays in COVID-19 vaccinations, with a vaccination rate of less than 4% until May 21, 2021 [8]. Only 25.4% of the population was fully vaccinated by the opening of the Olympics on July 23, 2021, arousing widespread suspicions of the safety of the Olympics [9]. Several previous studies indicated vaccine hesitancy as a key factor in early-stage vaccination in Japan [10,11].

Vaccine hesitancy is one of the main risks to world health reported by the World Health Organization (WHO) [12,13]. According to the 5 C model of vaccine hesitancy [14,15], the slow progress of early vaccination campaigns in Japan might have resulted from the inconvenience of vaccination and low vaccine confidence. The inconveniences of vaccination included lagged regulatory approval of COVID-19 vaccines, delayed vaccine importation, and a low-efficiency vaccine rollout system [8]. Japan ranked among the countries with the lowest vaccine confidence in the world [16], which might have stemmed from the crisis of confidence toward the human papillomavirus (HPV) vaccine in 2013 [17]. A deeper understanding of vaccine hesitancy during the slow progress of vaccination can be instructive for the COVID-19 booster vaccination and vaccinations against future pandemics.

Classical surveys are often used to gather data on public attitudes toward vaccination [18-20] but are often costly and time-consuming and only reflect relatively short-term situations and limited samples. In contrast, social media research can be cheaper and is more practical for collecting almost real-time information on the opinions and sentiments of a large population. During the pandemic, social media was used to mine opinions and sentiments toward the COVID-19 vaccine in various countries, but there remains a gap regarding Japan. Several studies have linked social media activity to vaccine hesitancy and antivaccine campaigns [21-30]. Tangherlini and colleagues [21] analyzed vaccine hesitancy drivers on blogs and reported that parents may utilize these forums to spread vaccine opposition views to other parents. Bonnevie and colleagues [24] studied the evolution of vaccine resistance in the United States by analyzing Twitter discussion topics and found that prominent Twitter accounts were responsible for a significant percentage of vaccine opposition messages. Lyu and colleagues [25] argued that public discussion is driven by major events and that vaccine sentiment around COVID-19 has become increasingly positive in 6 countries, showing higher acceptance rates than previous vaccines. The findings of several studies indicate positive attitudes in Australia, the United Kingdom, and the United States, but more positivity is needed to boost vaccination rates [26,27]. Marcec and Likic [28] examined the sentiment toward different vaccine brands and found that the sentiment in some countries toward the AstraZeneca vaccine seems to be decreasing over time, while sentiment toward the Pfizer and Moderna vaccines has remained positive and stable. Yousefinaghani and colleagues [29] conducted a large-scale study of people’s views of vaccines on Twitter. Chen and colleagues [30] summarized the topics of rumors on Twitter.

There were approximately 90.6 million Japanese social media users in 2020, accounting for 72.48% of the total population [31]. Moreover, the ongoing COVID-19 pandemic has led to the increasing use of social media as a venue for discussion of vaccine-related issues [32], and opinions of social media users may affect the opinions of others, resulting in vaccine hesitation or refusal [33].

This retrospective study aims to analyze the slow progress of early-stage vaccination in Japan by exploring the opinion and sentiment toward COVID-19 vaccines in Japanese tweets between August 1, 2020, and June 30, 2021, before and at the beginning of vaccination. We summarized the trends of the number of tweets and sentiments during the whole period. Regarding the reason for the slow vaccination process in Japan, the latent dirichlet allocation (LDA) topics of negative tweets since COVID-19 vaccination started were extracted. To investigate public attitudes toward different vaccine brands, sentiment and the top words of tweets related to 3 vaccine manufacturers were generated. The findings of this research can help governments, policy makers, and public health officials understand the factors that motivate and cause hesitance in the public toward vaccinations and provide evidence for planning, modifying, and implementing a tailored vaccine promotion strategy.

## Methods

### Data Extraction and Preprocessing

A large-scale COVID-19 Twitter chatter data set collected and maintained by Georgia State University’s Panacea Lab [34] was used in this study. The data set was still being updated and included 1.3 billion tweets by February 24, 2022. The data were collected through the publicly available Twitter Stream application programming interface (API) by querying the keywords “coronavirus,” “2019nCoV,” and “corona virus” in all available languages between January and March 2020, and the keywords were expanded to “COVD19,” “CoronavirusPandemic,” “COVID-19,” “2019nCoV,” “CoronaOutbreak,” “coronavirus,” and “WuhanVirus” after that. The tweet IDs, posting date and time, and languages of all tweets were gathered from the query results and provided in the data set. We first extracted the IDs of tweets marked as Japanese (some were mixtures of Japanese and English) and then collected the tweets using the Python package Tweepy. In this study, 1,305,308 tweets marked as being in the Japanese language from August 1, 2020, to June 30, 2021, were downloaded. It was noticeable that some tweets containing both English and Japanese were also marked as being in the Japanese language in the data set. All the English words in the tweets were transferred to half-width and lowercase. The tweets were filtered by keywords (Multimedia Appendix 1, Table 1) related to vaccinations. After data cleaning, 144,101 tweets with selected keywords were used for further analysis. Official data on the number of infections, deaths, and vaccinated cases were collected from the website of the Ministry of Health, Labor and Welfare (MHLW) [35] and the Prime Minister of Japan and His Cabinet (PMOJ) [36].

Tokenization is a fundamental step in many natural language processing (NLP) methods, especially for languages like Japanese that are written without spaces between words. We tokenized all tweets and analyzed the unigram and bigram tokens. The website links, special characters, numbers, and “amp” (ampersands) were removed from the tweets before tokenization. The Python packages SpaCy and GiNZA were used to remove the Japanese and English stop words and implement tokenization. The tokenized words were joined by white space characters into text in the original order. The Python package scikit-learn was used to convert the white space–joined texts into unigram and bigram tokens and to calculate the counts of tokens.

**Table 1:** Examples of tweets of different sentiments, paraphrased to protect user privacy.

Sentiment	Example
Positive	First vaccine! Muscle injection, surprisingly not painful.
Negative	I plan to get vaccinated, but in the absence of medium- and long-term verification, I remain concerned.
Neutral	Five vaccination sites are available for reservation in the Higashinari Ward.
Mixed	I heard that the Ministry of Education, Culture, Sports, Science and Technology will issue vaccination certificates for students planning to study abroad. That’s great, but isn’t the Ministry of Health, Labor and Welfare supposed to provide the vaccination certification? It's going to be confusing.

### Sentiment Analysis

Lacking efficient models and labeled corpora for a mixture of Japanese and English, we did not propose or fine-tune a model for sentiment analysis. Instead, Amazon Web Services (AWS) supporting multiple languages was chosen for this task. The sentiment analysis includes 4 labels: positive, negative, neutral, and mixed. The positive ratio of tweets was defined as the number of positive tweets divided by the number of negative tweets within the same time period. For each tweet, the model predicts the label and provides the score for each label. Some examples of tweets of different sentiments are in Table 1.

To determine the long-term tendency of the statistical data on public attitudes, we calculated the Pearson correlation coefficient (*r*) between the daily number of positive or negative tweets and the daily statistics for death, infection, and vaccinated cases using the Python package NumPy. The closer the absolute value of the *r* to 1, the stronger is the linear correlation between X and Y. In this study, we calculated the correlations before and after the start of vaccinations in Japan to determine whether vaccinations influenced the correlations.

### Latent Dirichlet Allocation

LDA is an unsupervised generative probabilistic model widely used in topic modeling [37]. LDA regards the documents in a corpus as generated from different topics, and each topic generates the documents following a Dirichlet distribution. We applied LDA modeling of vaccine-related tweets consistent with recent studies in other countries that included topic modeling [25,27]. We first generated the document-term matrix, which recorded the token frequencies in each tweet. All the tweets were put into a list, where each tweet was converted into unigrams and reconnected by spaces between neighboring tokens. A document-term matrix was generated on the reconnected tweets. Similar to [27], we also adopted the R package ldatuning [38] for topic number selection. The scores of 4 different metrics were calculated for the topic numbers from 2 to 50 (Figure S1 in Multimedia Appendix 2). The topic number with lower scores for the metrics of “Arun2010” [39] and “CaoJuan2009” [40] and higher scores for the metrics of “Griffiths2004” [41] and “Deveaud2014” [42] are more suitable for LDA modeling. In this study, “Deveaud2014” and “CaoJuan2009” reached the highest score on 6 topics. We built a 6-topic LDA model using the Python package scikit-learn for the tweets of negative sentiments from the first dose vaccination (February 17, 2021) in Japan to June 30, 2021. The results were made into bar plots of different topics to show the concerns during this period. The theme of each topic was summarized by 3 volunteers after a group meeting. The expectation of the number of tweets belonging to the *i*-th LDA topic was calculated by summing up the probability for each tweet generated by the *i*-th LDA topic.

### Peak Detection of Daily Trends

To provide an overview of the data, we plotted the trends of the daily number of total tweets and positive or negative tweets. For more precise analyses, the peaks in the data were labeled on the plots. For a human-like but objective selection, the peaks were determined by an algorithm with reference to the Weber-Fechner law [43] instead of human observation. The peaks of each month were selected using the following ratio:



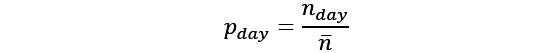



where *n_day_* is the number of tweets for the selected day and *n* is the average number of daily tweets in the month. If the ratio *p_day_* is higher than a threshold λ, the number of tweets of that day is judged as a peak. We then selected the valid peaks from all the peaks. Peaks with less than *n_min_* tweets and peaks within *d* days from the previous peak were discarded. In this study, λ=1.8, *d*=5, and *n_min_*=200 for the total number of tweets, and *n_min_*=40 for the number of positive or negative tweets. For all the detected peaks, we checked the tweets and provided the headline vaccine-related news in the tweets that day.

### Ethical Considerations

This study used publicly available and accessible tweets collected by Georgia State University’s Panacea Lab allowing free download. We assert that our analysis is compliant with Twitter's usage policy in aggregate form without identifying specific individuals who made the Twitter posts. Also, the numbers of infections, deaths, and vaccinated cases downloaded from the MHLW and PMOJ are open government data. Therefore, the activities described do not meet the requirements of human subject research and did not require review by an institutional review board.

## Results

### Overview of the Data

**Figure 1:**
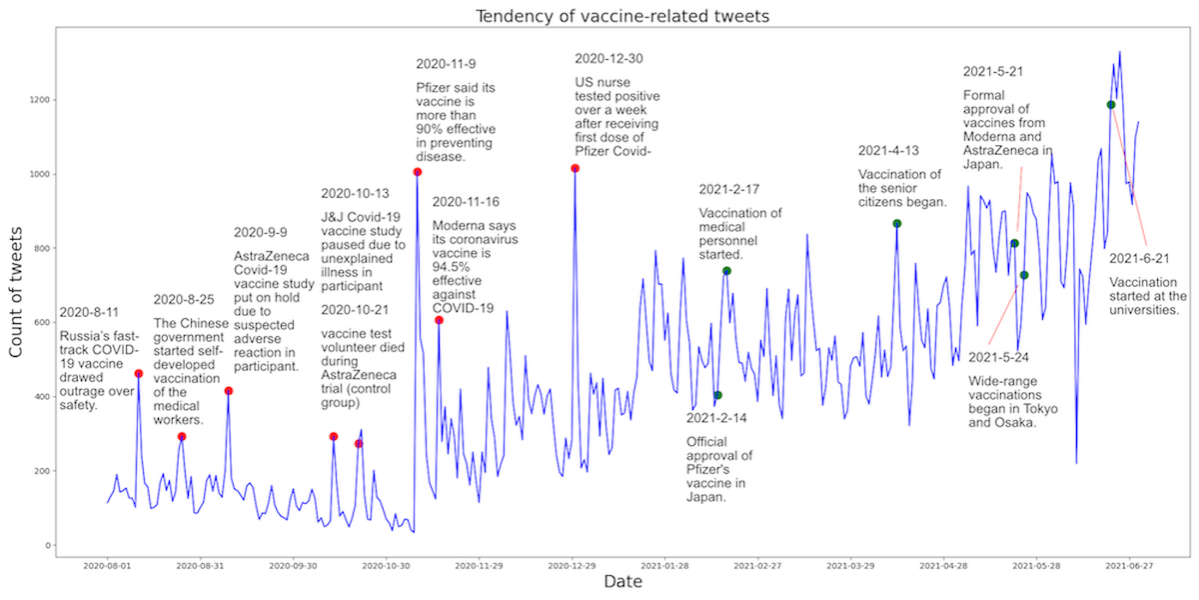
Trends of vaccine-related tweets with key events between August 1, 2020, and June 30, 2021. The red points indicate detected peaks, and the green points indicate important events related to vaccinations in Japan.

For a better understanding of the public attitude toward COVID-19 vaccines in Japan, we analyzed the trend and sentiments of vaccine-related tweets before and at the beginning of vaccination. We counted the number of vaccine-related tweets every day; the trends are shown in Figure 1. Headline news marking the milestones in Japan’s vaccinations were marked on the curve. The number of daily vaccine-related tweets increased continuously over the whole time period. Before November 9, 2020, the number of daily vaccine-related tweets was around or below 200. The detected peaks were related to some important vaccine-related news. On August 11, 2020 (n=463; n indicates the number of tweets), Russia approved the world’s first COVID-19 vaccine [44]. On August 25, 2020 (n=293), the Chinese government initiated the vaccinations of medical workers with self-developed vaccines. On September 9, 2020 (n*=*416), the AstraZeneca COVID-19 vaccine study was put on hold because of suspected adverse reactions in the participants. Two relatively small peaks (n= 292, n=274) were related to negative news about clinical trials in October 2020. The initial sharp peaks in the number of daily vaccine-related tweets were on November 9, 2020 (n=1006) and November 16, 2020 (n=606): Pfizer stated that its vaccine was 90% effective [5], and Moderna reported an effectiveness of 94.5%. The second surge in the number of daily tweets occurred on December 30, 2020 (n=1015), when a US nurse tested positive over a week after the first dose of vaccinations [45]. No additional peaks were detected in the following period, but the daily number of vaccine-related tweets continued increasing, especially after April 2021. This may be related to large-scale vaccinations in Japan, which is a long-term event across months.

**Figure 2:**
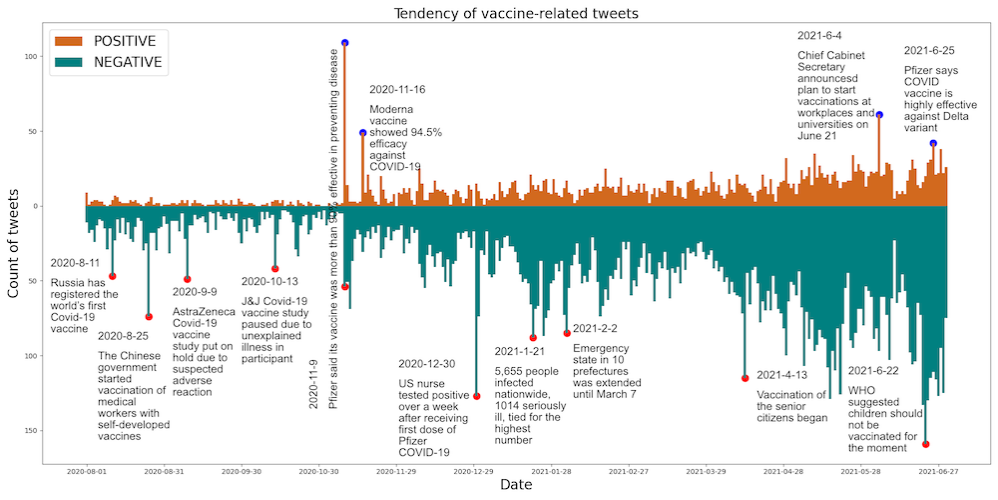
Trends of positive and negative sentiments between August 1, 2020, and June 30, 2021. The headline news related to the peaks are labeled.

We applied sentiment analysis to all tweets and counted the number of daily tweets for different sentiments in Figure S2 of Multimedia Appendix 2. About 85% of tweets displayed neutral sentiment, and negative sentiment overwhelmed positive sentiment in the other tweets. In Figure 2, we display the trends of positive and negative tweets with events. Negative sentiments mainly came from 3 aspects. The prompt response to vaccine rollout in other countries and news related to the side effects elicited negative public sentiment in Japan. Russia approved the first world’s COVID-19 vaccine (August 11, 2020; n=47); China started to administer its self-developed vaccine to medical workers on August 25, 2020 (n=74). Negative news about vaccinations in other countries also caused negative sentiment in Japan. AstraZeneca and Johnson & Johnson paused their studies on September 9, 2020 (n=49), and October 13, 2020 (n=42); a nurse in the United States was infected after the first dose of vaccine on December 30, 2020 (n=127); and the WHO suggested that children should not be vaccinated in the current stage on June 22, 2021 (n=159). Negative sentiment also came from the severe infection situation and the local vaccination policy in Japan. Cases of infection and serious illness both reached the highest on record on January 21, 2021 (n=88); 10 prefectures extended the emergency statement on February 2, 2021 (n=114), and senior citizens started to get vaccinations on April 13, 2021 (n=114). In contrast, the high efficiency of the vaccine and the start of large-scale vaccinations in Japan triggered positive sentiment. On November 9, 2020 (n=83), and November 16, 2020 (n=41), Pfizer and Moderna announced their vaccines’ effectiveness reached over 90% [46] and 94.5% [47], respectively, and on June 25, 2021 (n=42), a vaccine effect against the Delta variant was reported. On June 4, 2021 (n=61), the chief cabinet secretary announced the opening of vaccination appointments for workplaces and universities starting June 21, 2020. In addition, it is notable that the positive news about clinical trials also caused a peak of negative attitudes on November 9, 2020 (n=49).

### Correlation of Total Tweets and Positive or Negative Tweets With Cases, Deaths, and Vaccinations

**Figure 3:**
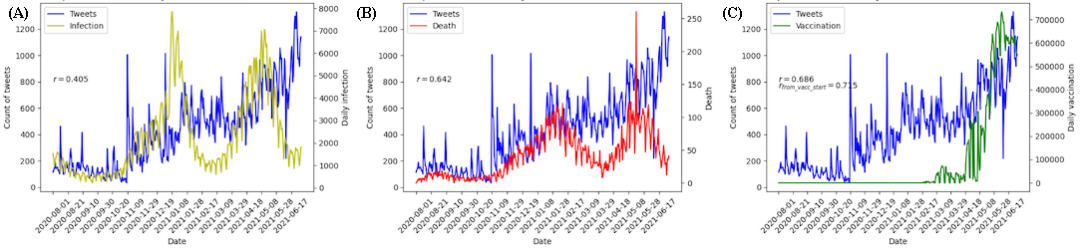
Trends of daily number of tweets with the daily numbers of (A) cases, (B) deaths, and (C) vaccinations.

**Figure 4:**
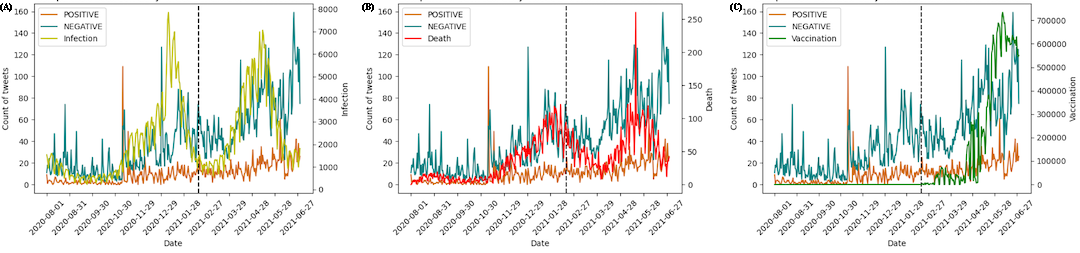
Trends of positive and negative sentiment together with the daily number of (A) cases, (B) deaths, and (C) vaccinations. The dashed line indicates the first day of vaccinations in Japan (February 17, 2020).

As shown in Figures 3 and 4, we calculated the correlation coefficients of total tweets and positive or negative tweets with the daily death, infection, and vaccinated cases, both before and after the first vaccination in Japan (February 17, 2020, dashed line in Figure 4). As Table 2 shows, the daily number of tweets showed correlations with the numbers of deaths, cases, and vaccinations (*r*=0.642, 0.405, and 0.686, respectively; *r*=0.715 after the first vaccination); negative sentiment was strongly correlated with deaths (*r*=0.691) and cases (*r*=0.500) before the first vaccination, but they later decreased to 0.305 and 0.293, respectively. The correlation between negative sentiment and vaccinations was slightly higher than the correlation with positive sentiment after the first vaccination.

**Table 2:** The correlations of positive and negative sentiment with daily deaths, cases, and vaccinations before and after the first vaccination in Japan.

Case types	Positive sentiment		Negative sentiment	
	Before, *r*	After, *r*	Before, *r*	After, *r*
Death cases	0.309	0.350	0.691^a^	0.305
Infection cases	0.242	0.147	0.500^a^	0.293
Vaccinated cases	N/A^b^	0.532^a^	N/A^b^	0.575^a^

^a^*r*≥0.5.

^b^The first vaccination in Japan was February 17, 2020, so we calculated the correlation before and after that day, respectively.

### Topic Modeling of Negative Tweets After Vaccination Started

**Figure 5:**
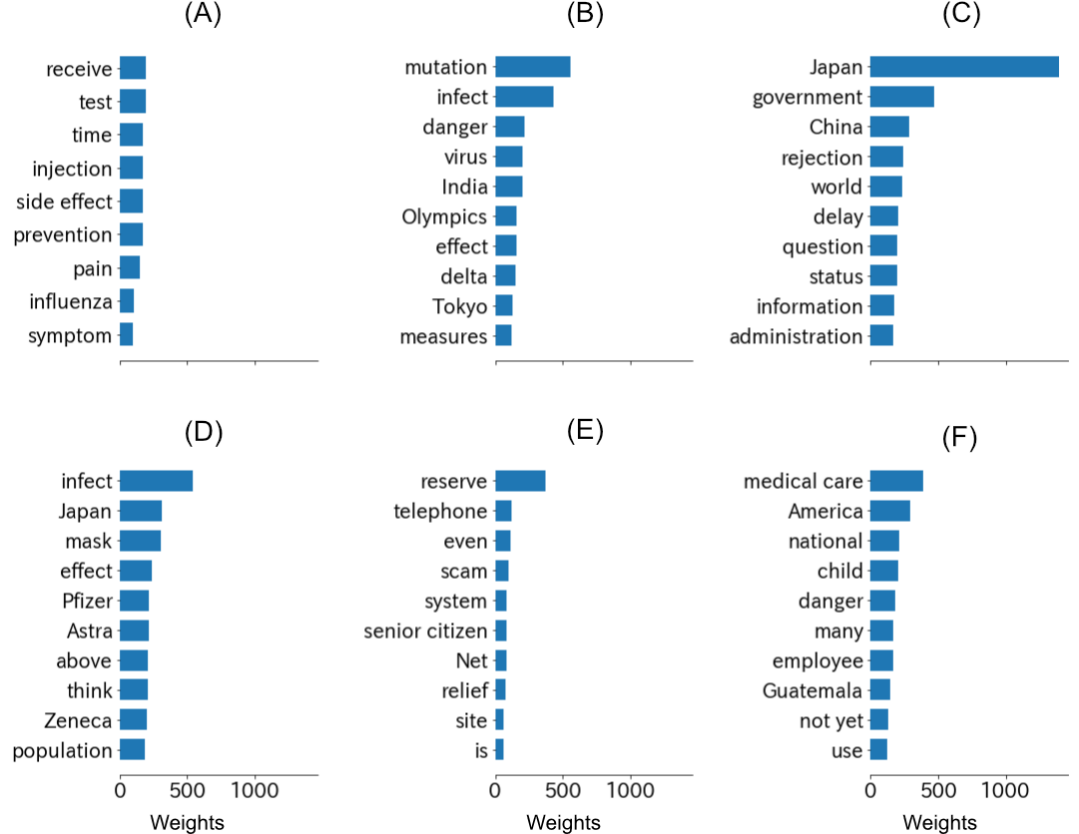
Top 10 keywords (translated into English) for each of the 6 topics of the latent dirichlet allocation (LDA) model built on negative tweets after vaccination started. The weights can be regarded as the pseudo counts of the keywords in each topic.

To analyze the problems at the early stage in Japan regarding public attitude, LDA topics were extracted from negative tweets after the first dose of vaccination in Japan. The top 10 keywords for each of the 6 LDA topics are shown in Figure 5. Topic 1 might be related to worries on the safety of vaccines. The theme of Topic 2 could be concerns about the risk of infection during the Tokyo Olympics. Topic 3 might show dissatisfaction of the public with the slow vaccination process in Japan compared with other countries. The top keywords in Topic 4 indicate discussions of the effectiveness of different vaccine brands. Topic 5 was related to the vaccine reservation system and telephone scams toward senior citizens during the vaccination process. Topic 6 indicated diffidence on the safety of the vaccines, especially regarding the injections of children and medical staff.

**Figure 6:**
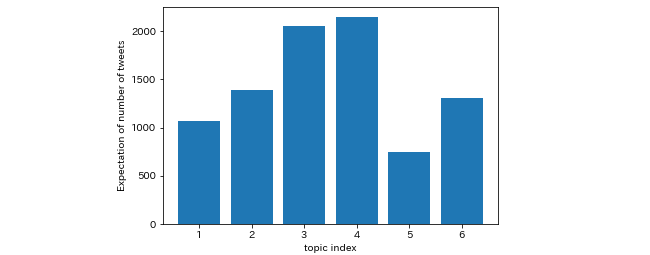
Expectation of number of tweets generated for each latent dirichlet allocation (LDA) topic.

The expectations of the numbers of tweets generated for each topic are shown in Figure 6. Expectations of the number of tweets generated by Topic 4 (2165) and Topic 3 (2062) were the highest, and the expectation of tweets generated by Topic 5 (815) was the lowest. The sum of the expectations of Topics 1 (1087) and 6 (1338) was slightly larger than that of Topic 4.

### Three Manufacturers’ Vaccines

**Figure 7:**
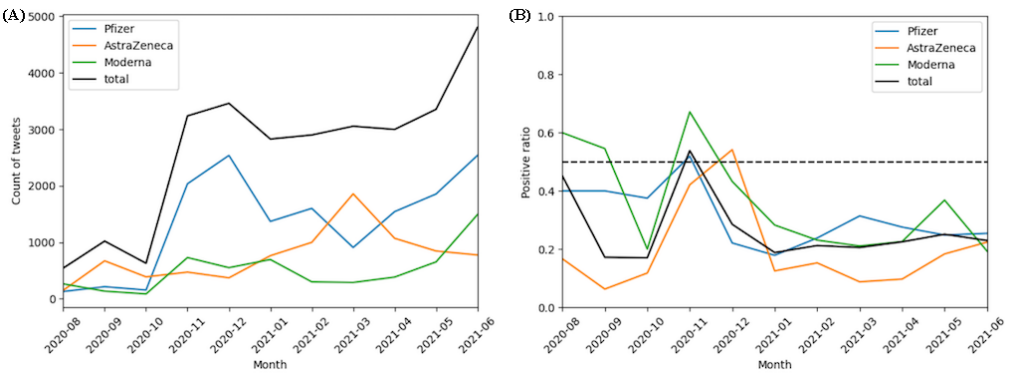
(A) Monthly number and (B) positive ratio of tweets related to the Pfizer, AstraZeneca, and Moderna vaccines between August 1, 2020, and June 30, 2021.

We analyzed the public attitudes toward the 3 vaccine brands approved by the Japanese government. The numbers of related tweets, in descending order, were 12,089 for Pfizer, 7300 for AstraZeneca, and 3338 for Moderna. As shown in Figure 7, public concerns about the 3 vaccines showed temporal variations. AstraZeneca vaccines showed an overall upward trend with peaks in September 2020 and March 2021 and a gradual decline after March 2021. The Pfizer vaccine received greater attention than the others from October 2020 to February 2021 and reached a peak in November 2020. Moderna appeared less in discussions, but since March 2021, the number of related tweets has continued to grow along with those for the Pfizer vaccine. As for the positive ratio of tweets related to the 3 manufacturers, the positive ratios for all 3 vaccines were lower than 0.5, meaning that the sentiment toward all the vaccine brands was negative. AstraZeneca had the lowest (0.19) average positive ratio, while Moderna had the highest (0.36).

**Figure 8:**
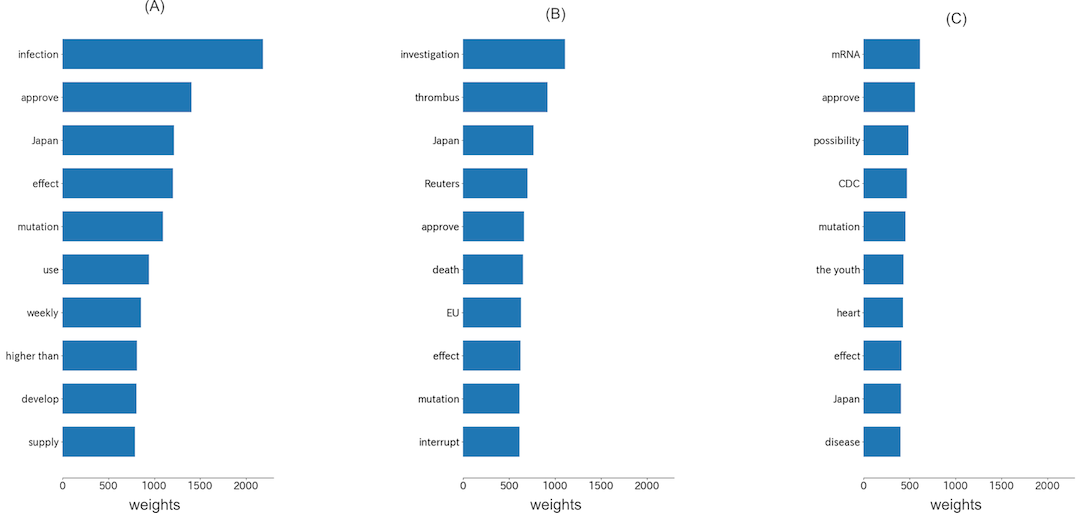
Top 10 words (translated into English) of tweets associated with the (A) Pfizer, (B) AstraZeneca, and (C) Moderna vaccines.

We analyzed the top 10 words in the tweets related to each vaccine brand. As shown in Figure 8, the discussion of all 3 vaccines mainly concerned effectiveness and side effects. According to the term frequencies of the top words for different manufactures, Pfizer was discussed more often than AstraZeneca and Moderna combined. There were also differences in the keywords of different vaccines. For Pfizer, the keywords were mainly about effectiveness and supplements. For AstraZeneca and Moderna, the top keywords also included severe side effects and thrombosis for AstraZeneca and heart disease for Moderna. It is also noticeable that “thrombus” ranked second in the top keywords of AstraZeneca.

## Discussion

### Principal Findings

This study examined long-term Japanese public opinion and sentiment, covering discussions from August 1, 2020, through June 30, 2021, when multiple vaccines were available yet only 13.6% of the population was fully vaccinated in Japan. We evaluated the trends and sentiments of vaccine-related tweets in Japanese. The overall number of tweets continued to increase after the start of large-scale vaccinations in Japan, which might have been primarily driven by critical events related to the vaccines. The sentiments of most tweets were neutral, with negative sentiment exceeding positive sentiment in volume. The correlations between the sentiments and the daily cases, deaths, and vaccinations were calculated. We also checked the LDA topics of negative sentiment since the vaccination started to identify the problems of vaccination at the early stage. Finally, we analyzed the trends in public sentiment about 3 vaccine brands (Pfizer, Moderna, and AstraZeneca), which showed a temporal shift as clinical trials moved forward, but whose core remained effective and secure. The top words for tweets related to each brand were also collected.

Our results show that negative sentiment outweighed positive sentiment in Japan, whereas most previous studies of other countries demonstrated more positive sentiment on social media [26,27,29]. The negative sentiment exhibited by our results is consistent with the findings of some previous survey studies in Japan [48-52], and we provided fine-grained and more practical evidence. We observed a decrease in the correlations of negative sentiment with numbers of cases and deaths in Table 2, for which the direct reason is the decrease in the numbers of cases and deaths and the abnormal increase in negative sentiment. The decrease in the numbers of cases and deaths may result from the effect of emergency statements and vaccinations, but the increase in negative sentiment might have stemmed from the frequent negative news about the messy process of vaccinations, including busy phone lines, website crashes, and incorrectly administered vaccinations in this period. We also found that the same event could trigger positive and negative sentiments in Japan. On November 9, 2020, when Pfizer reported that its vaccine was more than 90% effective, the peak of both positive and negative sentiments might indicate public expectation of its effectiveness and concerns about its safety. We presume that the negative sentiment was caused by the accumulation of negative news from different vaccines during clinical trials. There was a downward trend in both deaths and infections after the first dose, contrary to the trend of a consistent increase in the number of tweets.

The LDA topics of negative tweets reflected public concerns at the early stage of vaccination. Topics 1 and 6 concern the safety of vaccines. The sum of the expectations of the 2 topics was overwhelmingly greater than those of the other topics, which indicates that concerns about side effects might have outweighed the fear of infection at the beginning of the vaccination process. Similar results showing higher concerns about vaccine safety than the risk of infection can be found in classical surveys in other countries [53,54]. Topic 3 showed dissatisfaction with the slow vaccination process in Japan. This result is consistent with a survey-based study indicating disappointment of the Japanese public because of better performance during COVID-19 in neighboring countries [55]. Topic 2 showed pessimistic attitudes toward the Olympics, especially under the threat of a mutated virus, which is consistent with the results of a survey on the attitudes toward the Tokyo Olympics in several countries [9]. The expectation of the number of tweets generated by Topic 5 was the lowest, but the theme of Topic 5 directly indicated some problems during the vaccination process. The vaccine rollout system was inefficient at first in Japan [8], and people found it hard to reserve COVID-19 vaccines even though supplementation of vaccines was sufficient [56]. The reservation difficulties also provided a chance for telephone scams [57], which might have increased distrust in the vaccination process.

The sentiments and top words differed slightly across the vaccine brands, but the core remained effective and secure. Compared to the other 2 vaccines, the public tended to focus on the effectiveness of Pfizer in preventing infection, as opposed to Moderna, which tended to focus more on its effectiveness against mutated viruses and its mRNA development technology. The average percentage of positive sentiment for AstraZeneca was the lowest, similar to results in other countries [28]. The top words for both AstraZeneca and Moderna showed strong concerns among the public about the severe side effects of the vaccines. Furthermore, we found that “fake news” or misleading headlines caused public panic and widespread negative sentiment. For example, on October 21, 2020, numerous media outlets reported that the clinical trial of the AstraZeneca vaccine had led to the death of a Brazilian volunteer, which continued to trigger public panic even though the next day it was reported that the volunteer had not received the vaccine [58].

### Implications and Recommendations

The popularity of social media platforms coupled with NLP strategies benefits the government by enabling the monitoring of close-to-real-time public sentiment regarding vaccine information. This can inform more effective policy making and establish confidence toward vaccines so as to maximize vaccine uptake. Some of our findings provide new evidence and inspiration to academic researchers and can assist policy makers in capturing the relevant information needed in real time.

Our study provides Japanese public opinion and sentiment toward the COVID-19 vaccine with dynamic and unmodified expression. Japan ranked among the countries with the lowest vaccine confidence in the world, which might be linked to the HPV vaccine safety scares that started in 2013. However, the way in which the HPV vaccine scare was approached by health officials indicates continuing issues with the Japanese vaccination program that need resolving. Correspondingly, our findings indicate that the Japanese public showed significant negative sentiment before and at the beginning of vaccinations.

The LDA model of the negative tweets during the early stage of vaccination suggested some factors related to the slow vaccination process, which all indicated the importance of the swift action of the government and policy makers during the vaccination process. The strong concerns regarding the safety of vaccines at the beginning of the vaccination process suggest the necessity of building vaccine confidence. Swift and fair feedback on vaccine safety by policy makers could be especially important in the early stages of vaccination. Comparison with the vaccination process in other countries indicated the expectation of a prompt vaccination process in Japan. The claims regarding problems with the reservation system and vaccine-related telephone scams showed the urgency of building a convenient and safe pipeline for vaccine distribution and reservation, presenting the challenge of both the flexibility of the medical care system [8] and the acceleration of the digitalization process [59] in Japan. Concerns about the Olympics were also caused by the slow progress of anti-epidemic measures and the vaccination process but also showed the need for risk evaluation by policy makers balancing public health and other factors [60].

The public was concerned about the severe side effects of vaccines and was easily affected by fake or misleading news about the vaccines. To build vaccination confidence, it is also important to respond to concerns about the different vaccine manufacturers and respond to fake news in detail.

### Limitations

Our study has several limitations. First, as Twitter penetration is only 58.2 million (42.3% of the total population) people in Japan, our data may not be representative of the entire population, especially the older generations. Second, according to Twitter’s user privacy protection principles, our study could not further examine the demographic characteristics of users, such as age, gender, and geographic location. Third, due to the limitations of Japanese resources for sentiment analysis, we used AWS without training on data, so our findings might have been influenced by the accuracy of the model. Eventually, only a small number of people was vaccinated during our study period, especially older adults and health care workers. So, most of the tweets posted by users were based on information from the internet and news rather than direct experience with vaccinations. Accordingly, further in-depth studies are needed in the future.

### Conclusions

This study identified the Japanese public opinions and sentiments expressed on Twitter before and at the beginning of vaccination. The public attitude toward vaccination in Japan was negative, and the concerns about side effects might have outweighed the fear of infection at the beginning of the vaccination process, which reflected the necessity of boosting vaccine confidence. LDA topics of the negative tweets at the early stage of vaccination indicated that the government and policy makers should take prompt actions in constructing a safe and convenient vaccine reservation and rollout system, which requires both the flexibility of the medical care system and the acceleration of digitalization in Japan. People showed different attitudes toward the 3 vaccine brands. Policy makers should provide more evidence about the effectiveness and safety of vaccines and rebut fake news to build vaccine confidence.

## Appendix

Multimedia Appendix 1 These are translation tables between English and Japanese.

Multimedia Appendix 2 Supplementary figures.

## Abbreviations

API: application programming interface
AWS: Amazon Web Services
HPV: human papillomavirus
LDA: latent dirichlet allocation
MHLW: Ministry of Health, Labor and Welfare
NLP: natural language processing
PMOJ: Prime Minister of Japan and His Cabinet
WHO: World Health Organization
