# Supplementary material for: Public Opinion and Sentiment Before and at the Beginning of COVID-19 Vaccinations in Japan: Twitter Analysis
Source: JMIR Infodemiology. 2022 May 9;2(1):e32335. doi: 10.2196/32335 (PMC9092950; doi:10.2196/32335)

**Multimedia Appendix 2. Supplementary figures.**

**Figure S1** Plot of number of topics against LDA tuning scores for negative tweets since the first dose vaccination in Japan.


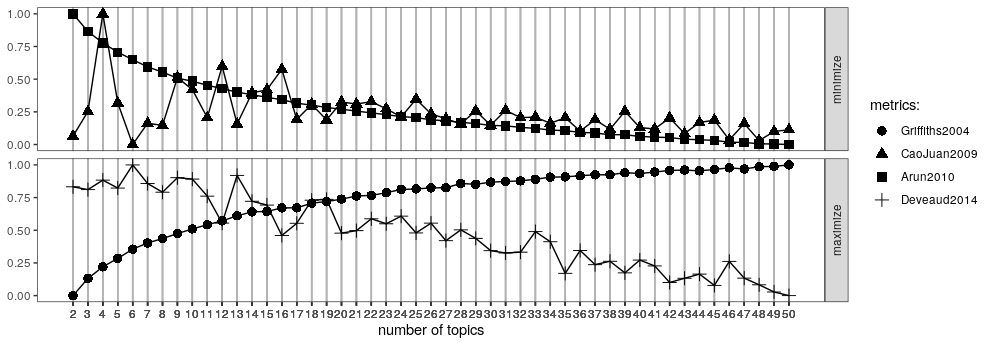


**Figure S2.** Trend of different sentiments between August 1, 2020, and June 30, 2021.


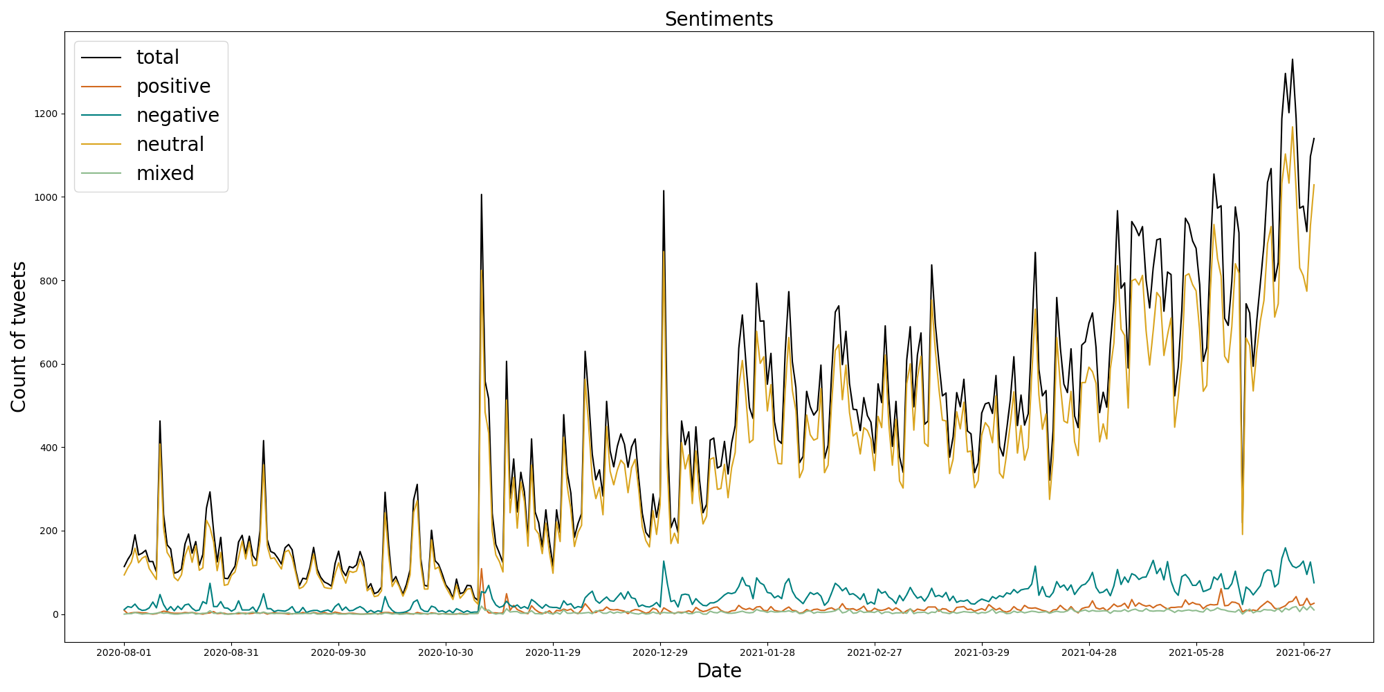

Supplement: Multimedia Appendix 2 [file infodemiology_v2i1e32335_app2.docx]
